# Proteomic analysis reveals an impaired Ca^2+^/AQP5 pathway in the submandibular gland in hypertension

**DOI:** 10.1038/s41598-017-15211-0

**Published:** 2017-11-06

**Authors:** Jing Zhang, Li-Jun Zhong, Yang Wang, Li-mei Liu, Xin Cong, Ruo-Lan Xiang, Li-Ling Wu, Guang-Yan Yu, Yan Zhang

**Affiliations:** 1 Department of Physiology and Pathophysiology, Peking University Health Science Center and Key Laboratory of Molecular Cardiovascular Sciences, Ministry of Education, Beijing, China; 2 Medical and Health Analysis Center, Peking University Health Science Center, Beijing, China; 3 Department of Oral and Maxillofacial Surgery, Peking University School and Hospital of Stomatology, Beijing, China

## Abstract

Hypertension is a systemic disorder that affects numerous physiological processes throughout the body. Improper sodium transport is a common comorbidity of hypertension, and sodium transport is also critical for maintaining the secretion of submandibular glands, whether the function of submandibular glands is affected by hypertension remains unclear. To determine whether hypertension induces changes in the protein expression of submandibular glands, we compared the proteome of submandibular glands from 14-week-old spontaneously hypertensive rats (SHR) and Wistar Kyoto (WKY) rats using LC-MS/MS. The results revealed that 95 proteins displayed different levels of expression between the submandibular glands from the SHRs and WKYs. Among these, 35 proteins were more abundant, and 60 proteins were less abundant in the SHR compared with the WKY rats. Specifically, aquaporin 5 and parvalbumin, which are correlated with water transport and intracellular Ca^2+^ signal transduction, were verified to exhibit differences in protein abundance. Impaired Ca^2+^ response to carbachol was confirmed in the acinar cells from SHRs, and hyposecretion by the submandibular glands was further confirmed by *in vivo* saliva collection. In conclusion, the proteomic analysis of the submandibular glands of SHRs revealed novel changes in protein abundance that provides possible mechanisms connecting hypertension and hyposecretion in submandibular glands.

## Introduction

Hypertension, also known as high blood pressure, is a long-term systemic disorder that affects numerous physiological processes throughout the body. Although many theories have been proposed to understand the pathogenesis and etiology of hypertension, in many cases, the detailed pathophysiology remain largely unknown^1^. Essential hypertension may be the result of increased sympathetic drive, a nonspecific increased response to norepinephrine, or abnormal cellular cation metabolism; specifically, Na^+^ imbalance may play a role in the structural reinforcement of hypertension^2^. Na^+^ reabsorption is enhanced in rats with various forms of experimental and genetic hypertension^3–5^. Moreover, Na^+^ transport plays an important role in regulating salivation^6^. Whether saliva production is affected by hypertension remains controversial. Elevated blood pressure has been reported to be associated with decreased salivary flow^7–10^. The infusion of angiotensin II into the parotid gland via the carotid artery causes a substantial reduction in the saliva secretion rate, which suggests a direct inhibitory effect of angiotensin II on the parotid that is possibly mediated by a constricting action on its vasculature or alters in water and electrolyte transport^11^. However, there are contradictory reports that have stated that there is no clear physiologic reason to suggest that hypertension should influence salivary function^12,13^. Therefore, whether hypertension affects salivation needs to be clarified.

The spontaneously hypertensive rat (SHR) is the most widely used animal model of essential hypertension and its accompanying metabolic disturbances when under special environmental conditions (for example, when fed a high-fructose or folate-deficient diet)^14^. Age-matched Wistar-Kyoto (WKY) rats are used as controls in most studies. Currently, the molecular changes that salivary glands undergo in chronic essential hypertension are not fully established.

The aim of this study was to apply a proteomic approach to identify altered proteins and pathways involved in salivation in the submandibular glands (SMGs) of SHRs compared with those in WKY. The results revealed altered protein abundances in the glands of the SHRs. We further confirmed an impaired Ca^2+^ signal, a reduced abundance of AQP5, and hyposecretion in the SHR SMGs.

## Results

### Protein expression analysis

**Figure 1:**
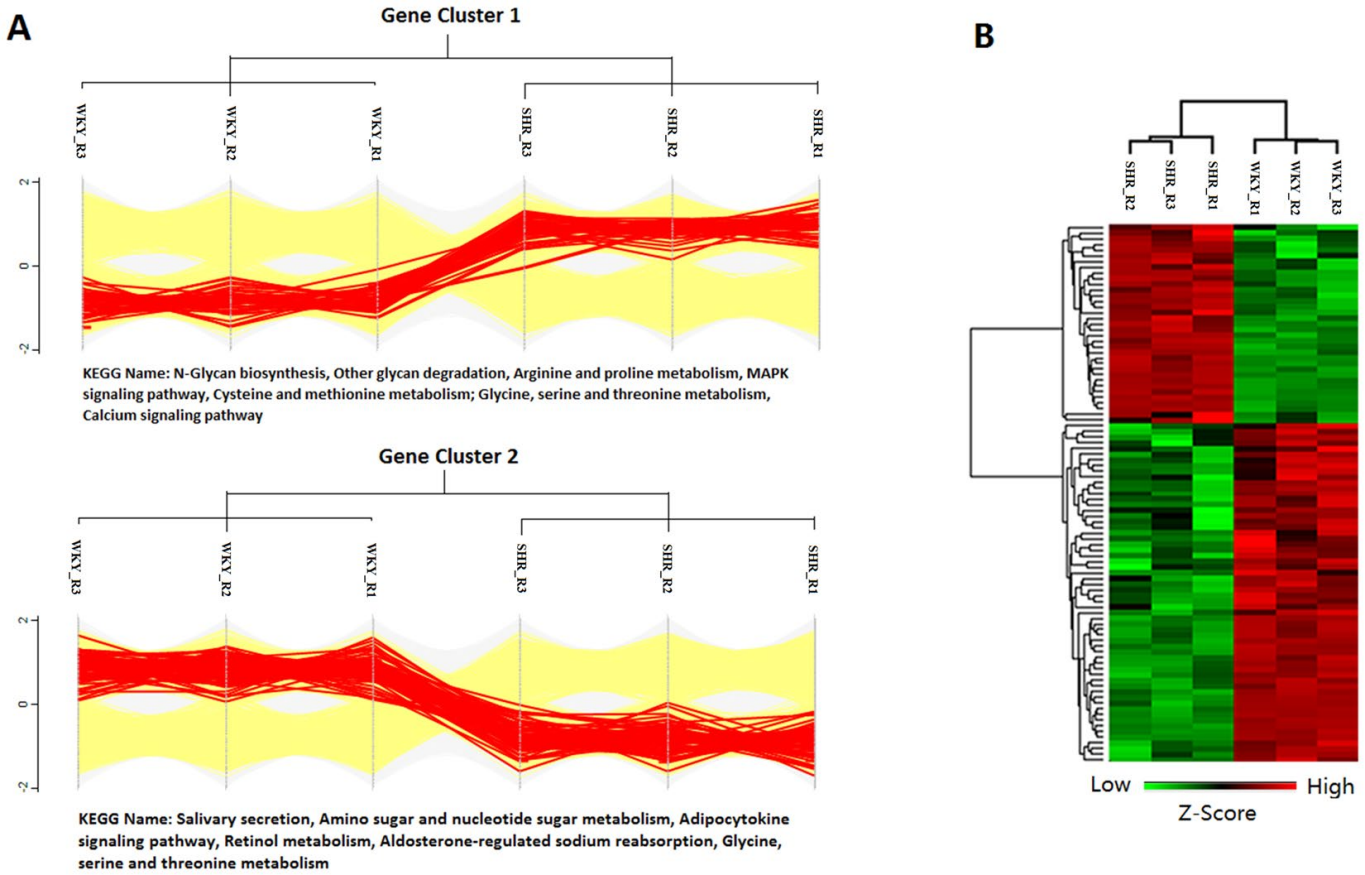
Analysis of the differentially expressed proteins. (**A**) Profile plot of the 95 differentially expressed proteins in 3 samples from each group. Categorical annotation in the form of participation in a KEGG pathway is supplied under the plot. (**B**) Hierarchical clustering analysis of the 95 significantly differentially expressed proteins. Red represents a high z-score, and green represents a low z-score.

Scatter plots of the log2-fold changes (X) and log10 *P* values (Y) revealed the proteome expression profiles of the two groups (Supplemental Fig. 1A). The red dots represent proteins that were up-regulated in the SHR group, and the green dots represent down-regulated proteins. The Venn diagrams represent the numbers of proteins that were identified in each of three biological replicates (Supplemental Fig. 1B). A total of 4619 proteins were identified in all three biological replicates using the shotgun method, and 4329 of these proteins were quantified with a label-free algorithm (at least 2 peptides of a protein were repeatedly measured in at least three samples). We found a total of 95 significantly differentially expressed proteins, among which 35 were up-regulated and 60 were down-regulated in the SHR group. To select the proteins that were differentially expressed in the SHR group, fold-changes greater than 2 and *P* values below 0.05 (using Student’s t-test) were used as the criteria. A profile plot of the differentially expressed proteins with categorical annotation (Fig. 1A) revealed that the up-regulated proteins in the SHR group were involved in the KEGG pathway of N-glycan biosynthesis, arginine and proline metabolism, a MAPK signaling pathway, cysteine and methionine metabolism, glycine, serine and threonine metabolism, and calcium signaling pathways, whereas the down-regulated proteins were involved in salivary secretion, amino sugar and nucleotide sugar metabolism, the adipocytokine signaling pathway, retinol metabolism, aldosterone-regulated sodium reabsorption, and glycine, serine and threonine metabolism. Hierarchical clustering analysis was performed to visualize the 95 significantly differentially expressed proteins between SHRs and WKYs. Red means up-regulated proteins and green means down-regulated proteins in SHR (Fig. 1B). The details for the top 10 proteins that were more or less abundant in the SHRs compared with the WKYs are listed in Table 1.

**Table 1:** Top 10 proteins more or less abundant in SHR compared with WKY.

Accession	Gene	Protein	Fold change	−log *P* value
***Less abundant in SHR***				
**P47864**	Aqp5	Aquaporin-5	0.092867715	3.13868
**D4AED6**	RGD1307782	Williams-Beuren syndrome critical region protein 27	0.095108757	2.89472
**M0RE00**	Tbca	Tubulin-specific chaperone A	0.12963352	3.46293
**Q63550**	Bpifa2f	BPI fold containing family A, member 2 F	0.159751431	3.09471
**G3V709**	Naprt1	Nicotinate phosphoribosyltransferase	0.167758024	4.62788
**P48450**	Lss	Lanosterol synthase	0.189945151	4.40373
**D4AAB9**	Paip2b	Poly(A) binding protein interacting protein 2B	0.201330033	1.55368
**Q5PQP2**	Ebag9	Receptor-binding cancer antigen expressed on SiSo cells	0.224552021	1.66317
**D3ZN19**	Rnf121	Ring finger protein 121	0.231378992	1.91671
**Q99JC6**	Tapbp	TAP binding protein	0.232509528	3.3854
***More abundant in SHR***				
**P02625**	Pvalb	Parvalbumin alpha	2.441750094	3.11535
**P08733**	Myl2	Myosin regulatory light chain 2, ventricular/cardiac muscle isoform	2.573624401	1.57846
**G3V885**	Myh6	Myosin-6	3.018308012	3.51078
**Q66H40**	Hmgn3	High mobility group nucleosome-binding domain-containing protein 3	3.033480339	1.93548
**Q4PP99**	Tnnc1	Troponin C type 1	3.082486958	2.58497
**O09171**	Bhmt	Betaine–homocysteine S-methyltransferase 1	3.765760717	5.04722
**P47727**	Cbr1	Carbonyl reductase 1	3.773230395	5.12838
**P13601**	Aldh1a7	Aldehyde dehydrogenase, cytosolic 1	5.507811394	3.67567
**D4A0S3**	Grpcb	Glutamine/glutamic acid-rich protein A	5.534638554	3.47886
**Q9QZ76**	Mb	Myoglobin	6.765551592	3.61407

### Decreased expression and membrane distribution of AQP5 in the SMGs of the SHRs

**Figure 2:**
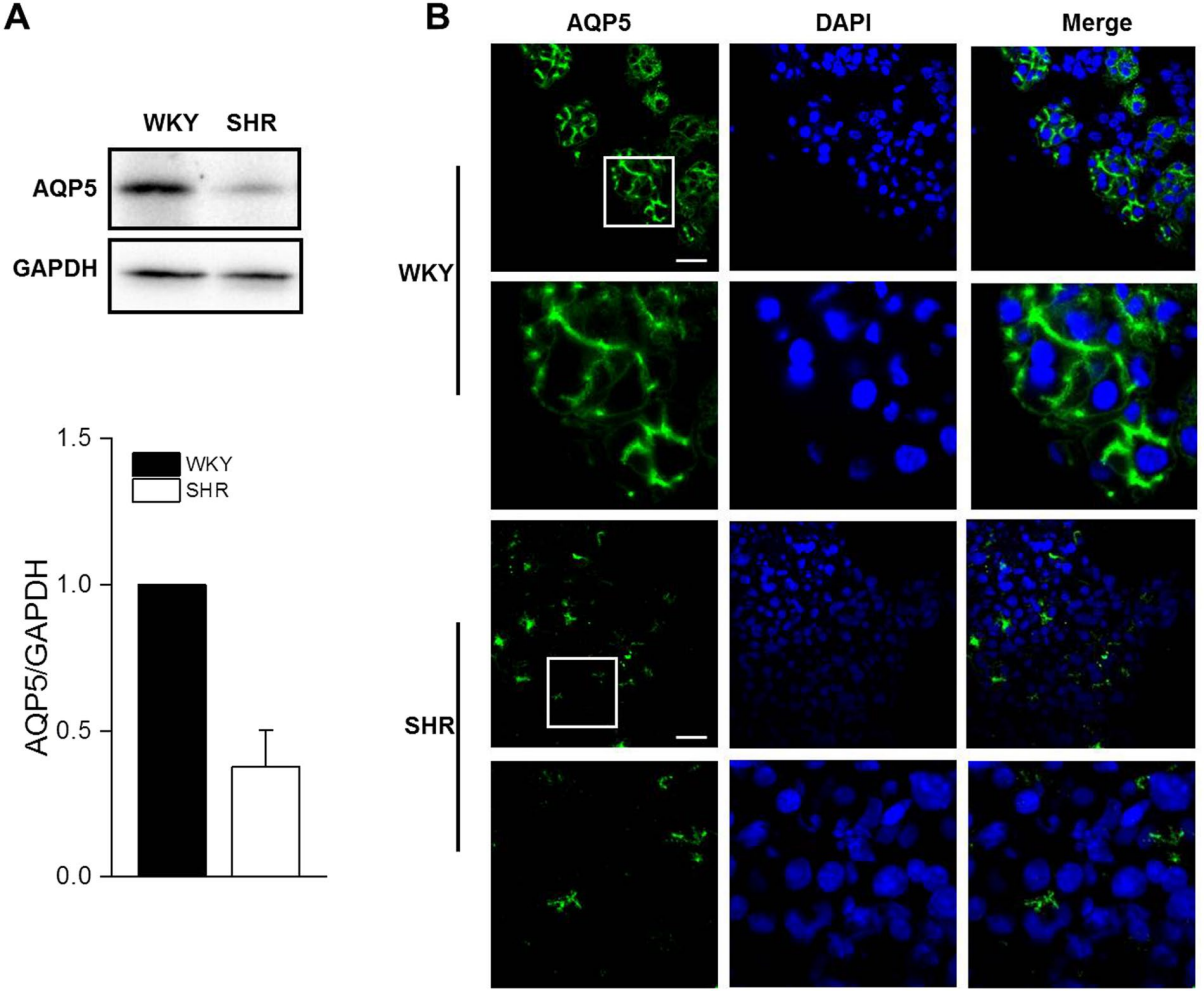
Decreased expression and membrane distribution of AQP5 in the SMGs of the SHRs. (**A**) Western blot analyses and (**B**) immunofluorescent staining for AQP5 in the submandibular glands of the SHR and WKY rats. The values are mean ± S.E.M. of four animals. **P < 0.01 vs. WKY. Scale bars: 30 μm. The displayed blots are cropped blots, and full-length blots are presented in Supplementary Figures 2 and 3.

Target proteins were chosen upon evaluation of the proteomics analysis (i.e., differential quantitation) and through consideration of a series of criteria that included protein-protein interaction analysis and the most relevant biological/molecular functions. For this purpose, one up-regulated protein (parvalbumin) and one down-regulated protein (AQP5) were chosen. Western blotting confirmed that AQP5 was obviously reduced in the SHR group compared with the WKY group (Fig. 2A). Immunofluorescent analysis revealed that AQP5 was mainly localized to the apical and lateral plasma membranes of the acinar cells in the WKY SMGs. In the SMGs of the SHRs, AQP5 staining in the acini was markedly declined, but the distribution still limited to the apical and lateral plasma membranes (Fig. 2B).

### Impaired calcium signaling in the SMGs of the SHRs

**Figure 3:**
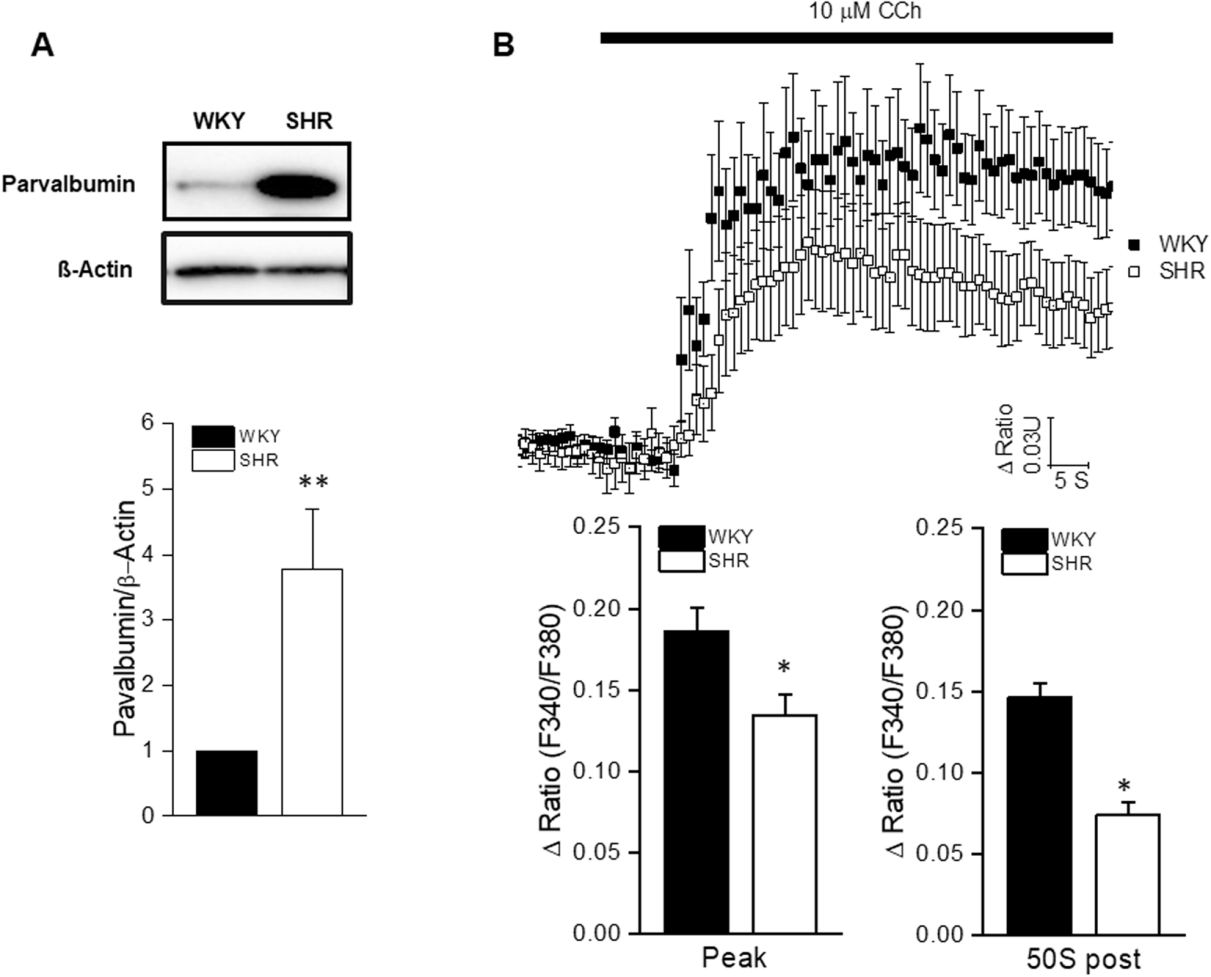
Impaired Ca^2+^ signaling in the SMGs of the SHRs. (**A**) Western blot analyses of parvalbumin in the submandibular glands of the SHR and WKY rats. The values are mean ± S.E.M. **P < 0.01 vs. WKY. The displayed blots are cropped blots, and full-length blots are presented in Supplementary Figures 4 and 5. (**B**) Acinar cells were loaded with fura-2 AM to determine the intracellular Ca^2+^ levels and were stimulated with 10 μmol/L carbachol during the indicated time. The magnitudes of the Ca^2+^ responses to carbachol at the peak and 50 s after stimulation are presented. The values are mean ± S.E.M. of 48 cells from six animals. *P < 0.05 vs. WKY.

Western blotting confirmed that parvalbumin protein expression was obviously reduced in the SHR group compared with the WKY group (Fig. 3A). Since parvalbumin acts as a Ca^2+^-binding protein and plays a role in Ca^2+^ homeostasis in many types of cells, whether the up-regulated parvalbumin altered the Ca^2+^ signal in the SMG needed to be studied. To clarify whether [Ca^2+^]_i_ mobilization changed in the SHRs compared with the WKYs, we measured the [Ca^2+^]_i_ in isolated SMG acinar cells in response to 10 μmol/L carbachol, which is an agonist of muscarinic receptors. The peak Ca^2+^ responses to carbachol and responses at 50 s after stimulation were much smaller in the SHRs than in the WKYs (Fig. 3B).

### Fluid secretion from the SMGs decreased in the SHR rats

**Figure 4:**
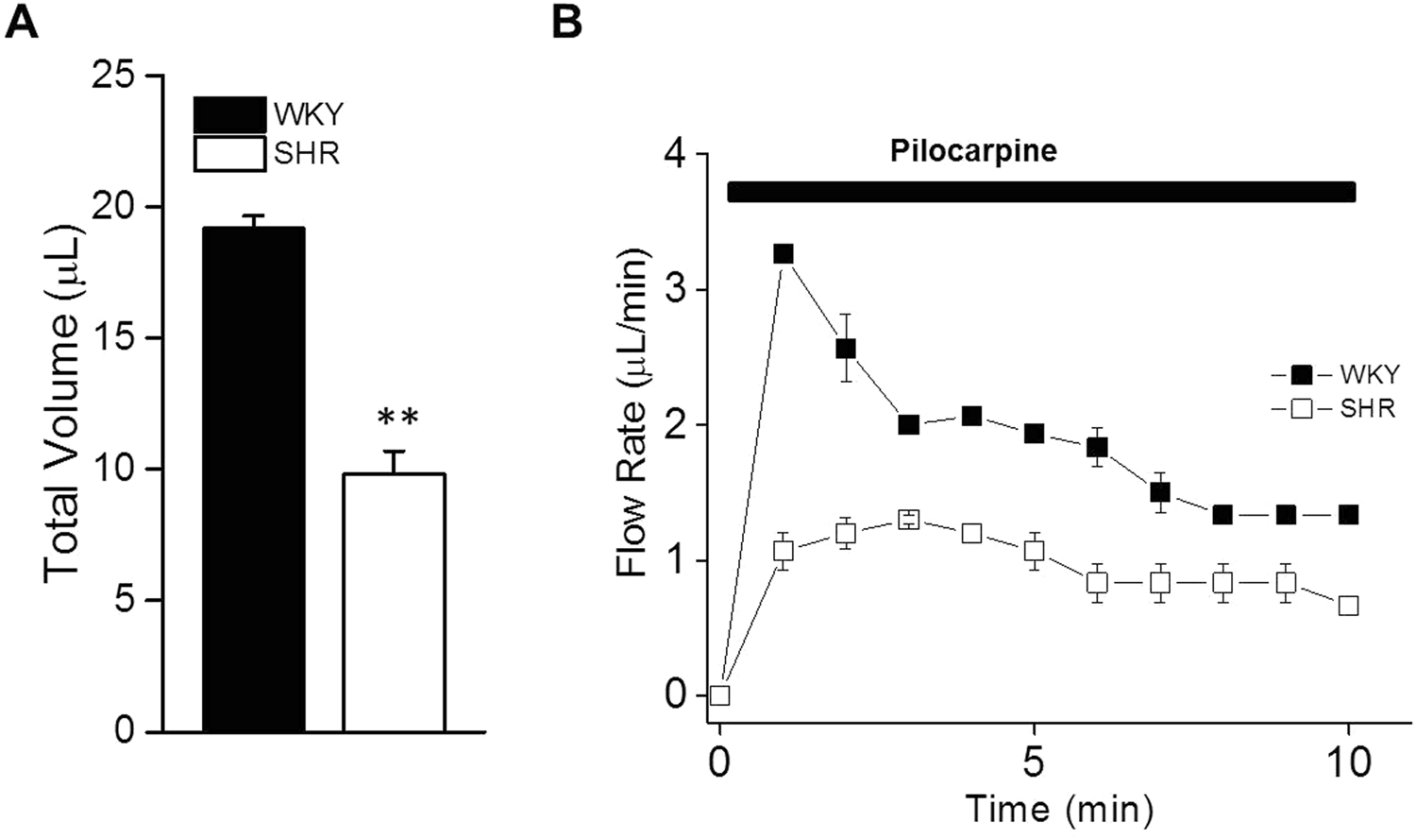
Changes in agonist-induced fluid secretion in the SHR and WKY rats. *In vivo* saliva collected from submandibular glands stimulated with pilocarpine (10 μg/g, i.p.). (**A**) The total volume (μl/10 min) and (**B**) flow rate (μl/min) were decreased in the SHRs compared with the WKYs. The values are mean ± S.E.M. of six animals. **P < 0.01 vs. WKY.

To investigate whether secretion by the SMGs was affected in the SHRs, *in vivo* saliva collection was performed from the SHR rats, and the WKY rats served as controls. The flow rates and total volumes of secreted saliva were measured in response to 10 μg/g pilocarpine. The total amount of saliva secreted in 10 min was decreased by 47.4% in the SHRs (Fig. 4A). The secretion rate was relatively constant but slower during the entire 10 min stimulation period in the SHR rats compared with the WKY controls (Fig. 4B). The *in vivo* saliva collections from the SMGs suggested impaired salivation in the SHR rats.

## Discussion

In the present study, we investigated the extent to which hypertension affected SMG protein expression and salivation. In total, 95 proteins exhibited expressions that were altered by over 2 fold in the SHR compared to the WKY rats. Furthermore, we demonstrated that secretion from the SMGs was decreased in the SHR rats. The mechanism included not only the down-regulated expression of AQP5 but also an impaired Ca^2+^ signal in the acinar cells. Accordingly, these results provide new insights into how hypertension may affect secretion from SMGs through alterations in the glands’ proteomes.

Clinical studies have reported that hypertension decreases saliva secretion in patients^7,9,10^. However, these studies have been challenged by other researchers who have found no obvious change in the flow rate in conditions of hypertension but have rather found potential pH changes^15^. Therefore, well-designed experiments are required to precisely investigate the effects of hypertension on salivary flow. Here, we conducted a proteomic investigation of the effects of hypertension on the SMG with the aim of identifying a possibly altered protein expression pattern in SHRs compared with WKYs using the techniques of LC/MS/MS. We found a total of 95 proteins with altered abundances in the hypertension group, and two of these that have been reported to be directly involved in the regulation of salivation were validated in subsequent experiments. The functions of the remaining 93 proteins need to be studied in the future.

AQP5 is well-known to be enriched in salivary glands, and in these glands, AQP5 is mainly localized in the apical membrane of the acinar cells and plays an important role in transepithelial water transport from the interstitium to the acinar lumen^16^. Proper expression and correct subcellular localization of AQP5 are required in maintaining salivary gland function^17–19^. Parasympathetic innervation is essential for the expression and distribution of AQP5 in the salivary gland^20–22^. In this study, we observed that AQP5 was localized to the apical and lateral plasma membranes of the acinar cells in the control SMGs, which is consistent with previous studies. However, in the SHR SMGs, AQP5 expression was dramatically decreased, and this decrease was among the most substantial in the proteomic analysis, which was validated using Western blot. The immunofluorescent results revealed that the staining pattern from the SHRs appeared similar to that from the WKYs. These results suggest that hypertension may decrease salivary secretion by directly down-regulating the expression of AQP5 without affecting its distribution.

Parvalbumins are acidic, intracellular Ca^2+^-binding proteins of low molecular weight. They are associated with several Ca^2+^-mediated cellular activities and physiological processes^23^. It has been suggested that parvalbumin might function as a “Ca^2+^ shuttle” that transports Ca^2+^ from troponin-C to the sarcoplasmic reticulum Ca^2+^ pump during muscle relaxation in muscle cells, which makes parvalbumin the most important soluble protein for Ca^2+^ buffering^24^. The overexpression of parvalbumin has been demonstrated to facilitate myocardial relaxation both *in vitro* and *in vivo* ^25^. The transfer of the parvalbumin gene into normal and diseased cardiac myocytes increases the relaxation rate but also markedly decreases the contraction amplitude^26^. Together, these results suggest that parvalbumin acts as a unique “delayed” Ca^2+^ buffer and facilitates Ca^2+^ sequestration from the cytosol. Additionally, the expression of parvalbumin is up-regulated in AQP4^−/−^ mice, which suggests that AQP4 affects intracellular Ca^2+^ dynamics by changing the expression of proteins involved in Ca^2+^ buffering^27^. In our results, the proteomic analysis revealed that parvalbumin was up-regulated in the SHR group, and this result was also validated by Western blot. Since the up-regulation of parvalbumin suggests a delayed Ca^2+^ signal in the SMG cells. We further detect the Ca^2+^ response to carbachol in isolated acinar cells from the SHR group, and the results demonstrated that the Ca^2+^ signal was impaired not only at the peak but also throughout the whole procedure when compared with the Ca^2+^ signal in the acinar cells of the WKYs. Whether parvalbumin was induced by impaired AQP5 in the SHRs is still unknown and requires further study.

In conclusion, our data demonstrated that protein abundances were significantly changed in the SMGs from the SHRs compared with the glands from the WKY rats. Among the proteins that were altered, AQP5 and parvalbumin were among those that exhibited the greatest changes in the proteomic analysis and were further validated. Secretion from the SMGs was obviously decreased in the SHR group. The underlying mechanisms of hyposecretion by the SMGs include not only the sequestration of Ca^2+^ from the cytosol by up-regulated parvalbumin but also the decreased expression of AQP5 in the glands. These findings improve our understanding of the mechanism by which hypertension suppresses saliva secretion and may provide new insights into future therapeutics for treating hypertension.

## Materials and Methods

### Animals

Male SHR (14 weeks old, early hypertension) and WKY (14 weeks old, normotension) rats were used in compliance with the Institutional Authority for Laboratory Animal Care of the Peking University Health Science Center, China. All experimental procedures were approved by the Ethics Committee for Animal Research of Peking University Health Science Center and complied with the Guide for the Care and Use of Laboratory Animals (NIH Publication No. 8523, revised 1996). A total of 16 rats in each group were used in this study. 6 rats were used in Ca^2+^ imaging, 6 rats were used in saliva collection, and 4 were used for Western blot, proteomic analysis, and immunofluorescence. The SMGs were carefully removed from the anesthetized rats and snap frozen in liquid nitrogen followed by storage at −80 °C.

### Chemicals

Ammonium bicarbonate, sodium deoxycholate, iodoacetamide, dithiothreitol, and all mobile phases and solutions prepared with HPLC grade solvents (i.e. water, acetonitrile, methanol, and formic acid) were purchased from Sigma (St. Louis, MO, USA). Tris-(2-carboxyethyl) phosphine was acquired from Thermo Scientific (Rockford, IL, USA). Modified sequencing-grade trypsin was obtained from Promega (Madison, WI, USA).

### Protein digestion and peptide fractionation

Crude protein was isolated from the SMGs and quantified as described previously^28^. The protein samples were digested according to the manufacturer’s protocol for filter-aided sample preparation^29^. The peptides were separated with high-pH reversed-phase chromatography using a Dionex Ultimate 3000 Micro Binary HPLC pump system^30^. The eluted peptides were pooled as 15 fractions and vacuum-dried. Next, the samples were ready for nano-ESI-LC-MS/MS analysis.

### LC-MS/MS and Label-Free Quantification

The analysis was performed in triplicate on a nano-flow HPLC system connected to a LTQ-Orbitrap Velos Pro mass spectrometer equipped with a Nanospray Flex Ion Source (Thermo Fisher Scientific, Waltham, MA, USA). Briefly, the peptides were separated in a pre-column (Easy-column C18-A1, 100 μm I.D.×20 mm, 5 μm) and a reversed phase C18 column (Easy-column C18-A2, 75 μm I.D. ×100 mm, 3 μm). The eluting peptides were ionized with an electrospray maintained at 2.2 kV, and the 15 most abundant ions detected in the full-MS scan were measured in the LTQ part by collision-induced dissociation. Each group was analyzed in three biological replicates. The raw data analyses were performed with MaxQuant software (version1.4.1.2, http://www.maxquant.org/). For protein identification, the MS/MS data were submitted to the Uniprot human protein database (release 3.43, 72,340 sequences).

Label-free quantitation (LFQ) was also performed in MaxQuant. The minimum ratio count for the LFQ was set to 2, and the match-between-runs option was enabled. The other parameters were set to the defaults. The up-regulated and down-regulated proteins were defined as those with significantly altered protein ratios and *P* values. The significance of the *P* values was calculated with the Perseus software (version 1.4.1.3, http://www.perseus-framework.org/).

### Bioinformatics Analysis

The protein expression analyses were performed with the Perseus software. Scatter plots were constructed with the log2-fold change (X) and log10 *P* value (Y). For the hierarchical clustering analysis of the differentially expressed proteins, the LFQ intensity of each protein was processed by a log2(x) transformation, and the Z-score was then calculated. The color of the bar represents the Z-score of each protein. The profile plot of the differentially expressed proteins was created with categorical annotation supplied in the form of the participation in a KEGG pathway. All annotations were extracted from the UniProt database. The Venn diagrams revealed the numbers of proteins identified in each of the three biological replicates.

### Proteomic validation by Western blotting

The proteins (40 μg) were separated on 12% SDS-PAGE gels, transferred to polyvinylidene difluoride membranes, blocked with 5% non-fat milk, probed with primary antibodies against aquaporin 5 (AQP5) (Santa Cruz Biotechnology, CA, USA) and parvalbumin (Abcam, Cambridge, MA, USA) at 4 °C overnight, and incubated with horseradish peroxidase-conjugated secondary antibodies. The immunoreactive bands were then visualized by enhanced chemiluminescence. ß-actin (Santa Cruz Biotechnology, CA, USA) and GAPDH (Abcam, Cambridge, MA, USA) was used as a loading control.

### Immunofluorescence

The glands were taken from −80 °C, embedded into OCT and cut into 5 μm sections. Frozen sections were fixed in cold acetone for 15 min, blocked with 10% goat serum, immunostained with primary antibodies against AQP5 (1:100) overnight at 4 °C followed by an Alex 488-labeled secondary antibody. The nuclei were stained with 4,6-diamidino-2-phenylindole (Sigma, St. Louis, MO, USA). The fluorescence images were captured by confocal microscopy (Olympus FV1200, Japan).

### Ca^2+^ Imaging

SMG acinar cells were prepared by enzyme digestion as previously reported with minor modification^31^. In brief, the rats were euthanized, and the glands were dissected, finely minced, digested in minimal essential medium (MEM, Invitrogen, Grand Island, NY, USA) containing 1% bovine serum albumin and 0.125 mg/ml Liberase TL enzyme (Roche Applied Science, Indianapolis, IN, USA) for 25 min at 37 °C, and then centrifuged. The cell pellet was resuspended and digested in Liberase TL for another 25 min. Following digestion, the cells were rinsed in MEM supplemented with 1% BSA.

The fura-2 fluorescence ratiometric method was used to measure the intracellular free calcium levels. The cells were loaded with 2 µM fura-2/AM for 25 min at room temperature. Imaging was performed using an inverted microscope (IX71, Olympus, Japan) equipped with a Polychrome IV Imaging System coupled to a high-speed digital camera (Till Photonics, Pleasanton, CA, USA). Images from the fura-2 (Invitrogen, Grand Island, NY, USA)-loaded cells were acquired every second for carbachol (Sigma, St. Louis, MO, USA) by alternate excitation with light at 340 nm and 380 nm, and emission was captured at 510 nm using MetaFluor7.0 (Molecular Devices, Sunnyvale, CA, USA). The chamber volume was maintained at ~100 µl. The fluorescence ratio of 340 nm over 380 nm was calculated, and all data are presented as the change in ratio units.

### Measurement of stimulated salivary flow

The rats were fasted for a minimum of 5 h with water provided ad libitum. The saliva secreted from the SMGs was collected for 10 min from surgically isolated ducts using a micropipette starting 5 min after pilocarpine (Abcam, Cambridge, MA, USA) injection (10 μg/g body weight, i.p.) under anesthesia. The volume of saliva was measured, and the salivary flow rate is represented as microliters per minute.

### Statistics

The data are expressed as mean ± S.E.M. The statistical analyses were performed using one-way ANOVA followed by Bonferroni’s test for multiple comparisons with Origin 2016 software (OriginLab, MA, USA). Differences were considered statistically significant at *P* < 0.05. For the discovery stage, a 2-fold change and a Student’s t-test *P* value of 0.05 were used as combined thresholds to define the biologically regulated proteins.

## Electronic supplementary material

Supplementary table and figures
