# Supplementary material for: Proteomic analysis reveals an impaired Ca2+/AQP5 pathway in the submandibular gland in hypertension
Source: Sci Rep. 2017 Nov 6;7:14524. doi: 10.1038/s41598-017-15211-0 (PMC5674029; doi:10.1038/s41598-017-15211-0)
Supplement: Supplementary file 1 — Supplementary table and figures [file 41598_2017_15211_MOESM1_ESM.doc]

**Proteomic analysis reveals an impaired Ca^2+^/AQP5 pathway in the submandibular gland in hypertension**

Jing Zhang^1#^, Li-Jun Zhong^2#^, Yang Wang^3^, Li-mei Liu^1^, Xin Cong^1^, Ruo-Lan Xiang^1^, Li-Ling Wu^1^, Guang-Yan Yu^3^, and Yan Zhang^1 *^

1 Department of Physiology and Pathophysiology, Peking University Health Science Center and Key Laboratory of Molecular Cardiovascular Sciences, Ministry of Education, Beijing, China

2 Center of Medical and Health Analysis, Peking University Health Science Center, Beijing, China

3 Department of Oral and Maxillofacial Surgery, Peking University School and Hospital of Stomatology, Beijing, China

^#^These authors contributing equally to this article.

^*^Corresponding author:

Yan Zhang, Department of Physiology and Pathophysiology, Peking University Health Science Center, 38 Xueyuan Road, Haidian District, Beijing, 100191, China. E-mail: zhangy18@bjmu.edu.cn

**Supplementary Table 1. P**roteins more or less abundant in SHR compared with WKY.

| Gene | Accession | Fold change | -log *P* value |
| --- | --- | --- | --- |

| ***Less abundant in SHR*** |  |  |  |
| --- | --- | --- | --- |
| Aqp5 | P47864 | 0.092867715 | 3.13868 |
| RGD1307782 | D4AED6 | 0.095108757 | 2.89472 |
| Tbca | M0RE00 | 0.12963352 | 3.46293 |
| Bpifa2f | Q63550 | 0.159751431 | 3.09471 |
| Naprt1 | G3V709 | 0.167758024 | 4.62788 |
| Lss | P48450 | 0.189945151 | 4.40373 |
| Paip2b | D4AAB9 | 0.201330033 | 1.55368 |
| Ebag9 | Q5PQP2 | 0.224552021 | 1.66317 |
| Rnf121 | D3ZN19 | 0.231378992 | 1.91671 |
| Tapbp | Q99JC6 | 0.232509528 | 3.3854 |
| Faf1 | F1LSQ0 | 0.268990929 | 2.05996 |
| Commd5 | Q9ERR2 | 0.272858881 | 2.0893 |
| Ocm | P02631 | 0.283967403 | 3.69491 |
| Qsox2 | D3ZP13 | 0.296468512 | 3.24538 |
| Ppp1r9a | O35867 | 0.308358342 | 3.00565 |
| RGD1565355;Cd36 | Q6IMX5 | 0.312576629 | 2.11896 |
| Oas1a | G3V9A4 | 0.31395229 | 2.89578 |
| LOC100363408;Bola2 | D4A9P7;D3ZVA6 | 0.318834477 | 2.57018 |
| Gstm1 | G3V983 | 0.320144874 | 4.15234 |
| Acsf2 | Q499N5 | 0.333513705 | 5.4367 |
| Gne | O35826 | 0.335548207 | 4.91537 |
| Ormdl2 | D4A2I4 | 0.339467156 | 1.41807 |
| Aox3 | Q5QE80 | 0.340061751 | 3.2658 |
| Klc2 | B2GV74 | 0.343925925 | 1.6827 |
| Sh3kbp1 | Q925Q9-7 | 0.345303486 | 2.43916 |
| Mx1 | P18588 | 0.349781764 | 4.32618 |
| Rabl3 | D4A1C1 | 0.355140605 | 1.95748 |
| LOC100909580 | G3V7N6 | 0.357777239 | 2.21892 |
| Abhd13 | D4A1B6 | 0.362889801 | 2.01844 |
| Gstm3 | P08009 | 0.386320597 | 3.06493 |
| Casp7 | F7F246 | 0.404246676 | 2.4938 |
| LOC100911374 | D3ZCR9 | 0.405684864 | 1.90836 |
| Mx2 | P18589 | 0.409430488 | 3.77491 |
| Retsat | Q8VHE9 | 0.41086434 | 1.5937 |
| Tmem201 | D3Z972;D3ZBW3 | 0.418308662 | 4.81235 |
| Rab2a | F1LP82 | 0.418710291 | 2.58351 |
| Endod1 | D3ZIP8 | 0.422708448 | 3.95058 |
| LOC298795;Sfn | Q5EBB0;G3V9A3 | 0.433193345 | 1.35109 |
| Cbfb | Q66HA7 | 0.434629902 | 2.34534 |
| Ca5b | Q66HG6 | 0.435773881 | 2.38393 |
| S100b | P04631 | 0.450749429 | 1.55464 |
| Mt-co1;Mtco1 | Q8SEZ6 | 0.451071148 | 2.49165 |
| Gnmt;LOC100911564 | P13255 | 0.451983263 | 2.07513 |
| Ddrgk1 | D3ZAS9 | 0.457008637 | 1.63878 |
| Sdcbp | Q9JI92 | 0.459962732 | 3.85595 |
| Inppl1 | Q9WVR3 | 0.463793486 | 1.74493 |
| Fam169a | D3ZKX8 | 0.46430203 | 1.60918 |
| Mrpl4 | D4A131 | 0.472322034 | 2.2098 |
| Psmb10 | Q4KM35 | 0.473544612 | 2.4611 |
| Mrpl44 | Q4G067 | 0.476857447 | 1.68939 |
| Csk | P32577 | 0.478791033 | 2.09719 |
| Casd1 | M0R6Q0 | 0.480432778 | 2.47356 |
| Napg | D4A0E2 | 0.480591456 | 1.85428 |
| Padi2 | P20717 | 0.486437724 | 3.54519 |
| Lgals9 | Q6P7Q6 | 0.487773053 | 2.35693 |
| Fam115c;LOC100912372 | M0RBL7;D3ZEW3 | 0.492409753 | 2.77774 |
| Bax | G3V8T9 | 0.493446117 | 2.79253 |
| Tiprl | A2VCX1 | 0.496506016 | 1.31661 |
| Aqp1 | P29975 | 0.498119497 | 3.17449 |
| Cdc123 | Q62834 | 0.499799467 | 2.59982 |
|  |  |  |  |
| ***More abundant in SHR*** |  |  |  |
| Orm1 | P02764 | 2.027959621 | 2.39037 |
| Mfap2 | D3Z952 | 2.037848671 | 1.82257 |
| Mgat4a | Q5M854 | 2.053428044 | 3.7338 |
| Tmcc3 | D3ZLE2 | 2.060850835 | 2.68957 |
| Man2c1 | F1LPQ3 | 2.060919765 | 3.2235 |
| Aldh18a1 | D3ZIE9 | 2.071928427 | 4.22982 |
| Cd200 | A0A5D0 | 2.081234445 | 3.75621 |
| Sult1d1 | G3V9R3 | 2.0813945 | 3.16871 |
| Serping1 | Q6P734 | 2.099729589 | 2.55239 |
| Ttr | P02767 | 2.1797491 | 2.19354 |
| Col4a2 | F1M6Q3 | 2.186241086 | 4.16715 |
| Col4a1 | F1MA59 | 2.222353052 | 3.5202 |
| Nqo1 | P05982 | 2.233216441 | 2.58242 |
| Arpp19 | Q712U5-2 | 2.266971882 | 2.98686 |
| Adhfe1 | Q4QQW3 | 2.294857143 | 4.69923 |
| Inmt | D3ZNJ5 | 2.395126552 | 3.81169 |
| Recql | Q6AYJ1 | 2.417174679 | 2.16994 |
| Pip | G3V812 | 2.428415501 | 3.67507 |
| Serpina3n | P09006 | 2.428841651 | 2.23053 |
| As3mt | Q8VHT6 | 2.428985671 | 3.49121 |
| Me2 | D3ZJH9 | 2.438014536 | 1.71967 |
| Slc25a16 | P16261 | 2.438229535 | 1.36802 |
| Bles03 | Q566Q8 | 2.438341578 | 2.34864 |
| Dusp22 | D3ZC16 | 2.439416209 | 4.16472 |
| Ephx2 | Q5RKK3 | 2.441068177 | 2.75021 |
| Pvalb | P02625 | 2.441750094 | 3.11535 |
| Myl2 | P08733 | 2.573624401 | 1.57846 |
| Myh6 | G3V885 | 3.018308012 | 3.51078 |
| Hmgn3 | Q66H40 | 3.033480339 | 1.93548 |
| Tnnc1 | Q4PP99 | 3.082486958 | 2.58497 |
| Bhmt | O09171 | 3.765760717 | 5.04722 |
| Cbr1 | P47727 | 3.773230395 | 5.12838 |
| Aldh1a7 | P13601 | 5.507811394 | 3.67567 |
| Grpcb | D4A0S3 | 5.534638554 | 3.47886 |
| Mb | Q9QZ76 | 6.765551592 | 3.61407 |


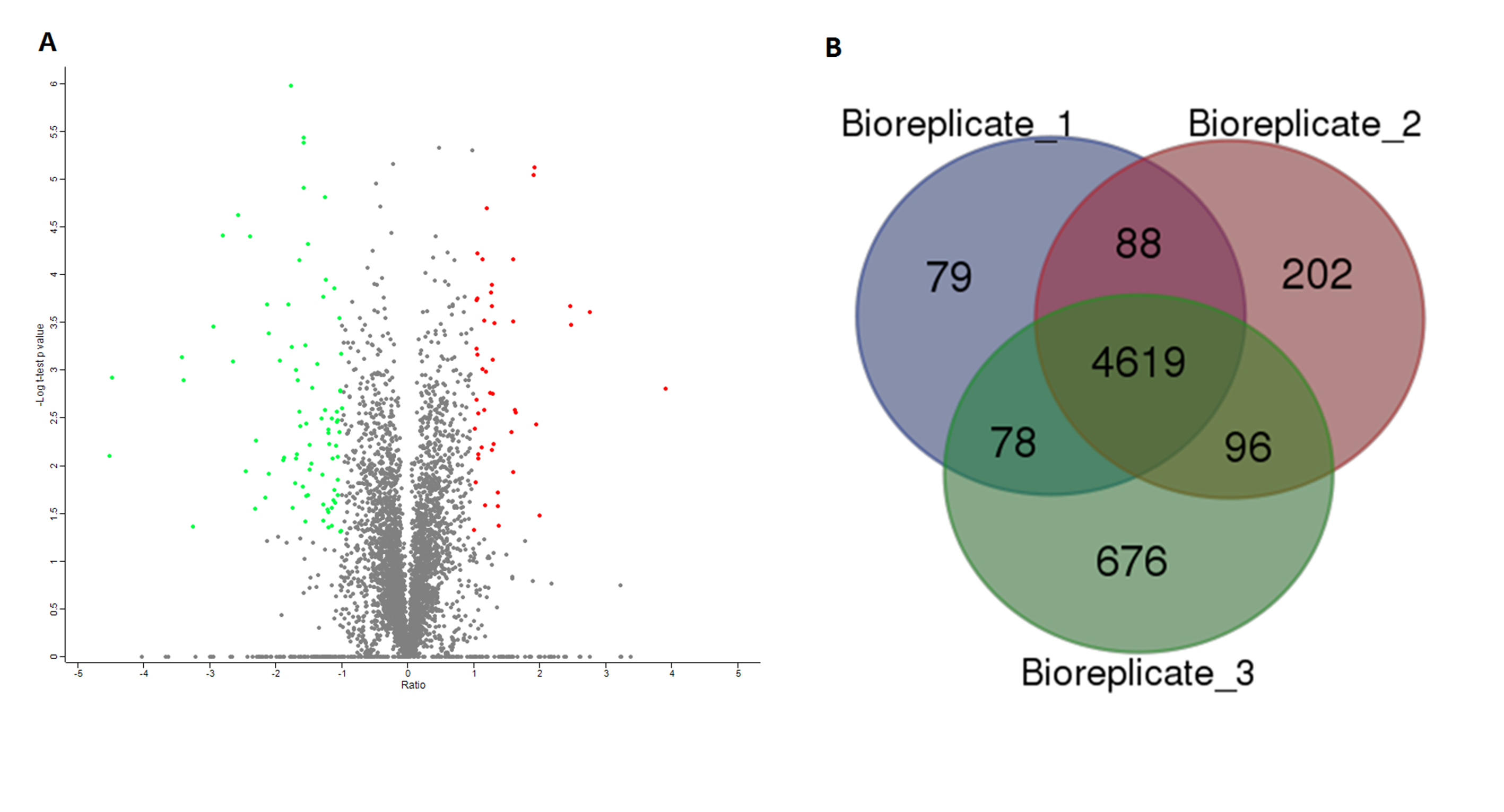


**Supplemental Figure 1.** Proteome expression analysis. (A) Scatter plots constructed with log2 fold change (X) and log10 *P* value (Y) of all identified proteins. (B) Venn diagrams show the number of proteins identified in each of the three biological replicates.


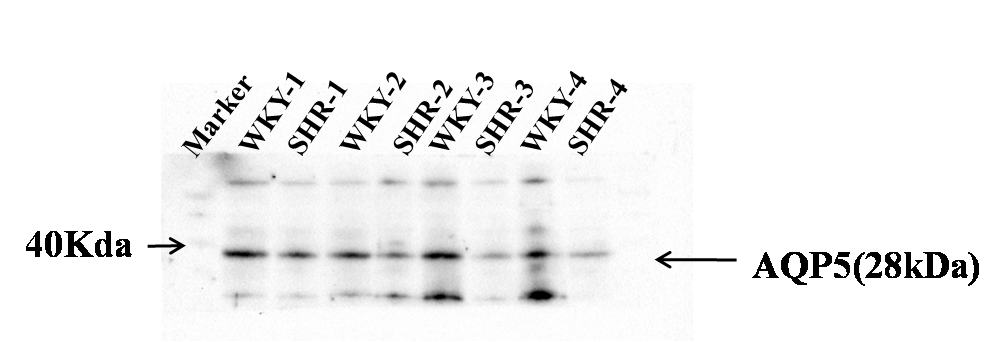


**Supplemental Figure 2.** Western blot analysis of AQP5 in the submandibular glands in SHR and WKY rats.


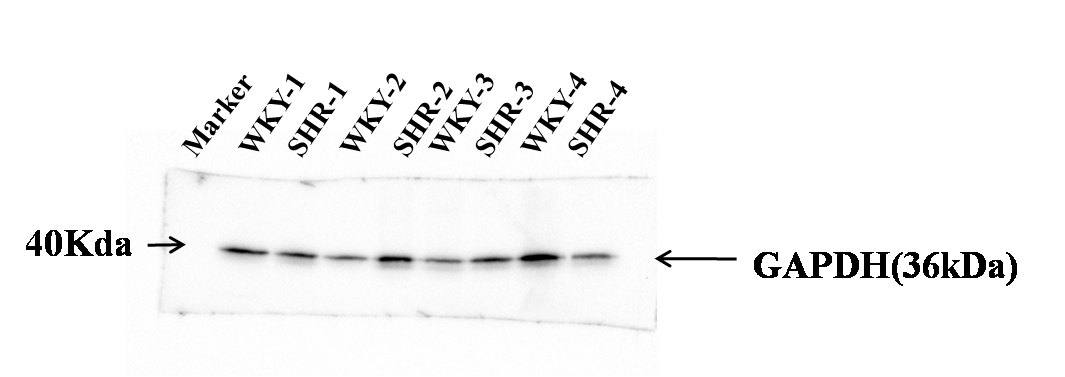


**Supplemental Figure 3.** Western blot analysis of GAPDH in the submandibular glands in SHR and WKY rats.


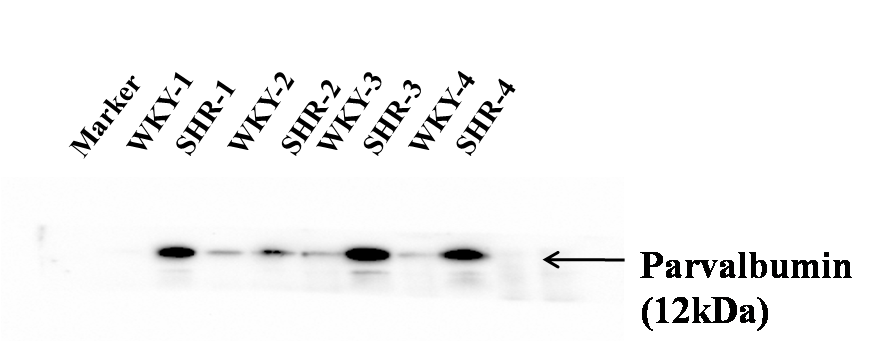


**Supplemental Figure 4.** Western blot analysis of parvalbumin in the submandibular glands in SHR and WKY rats.


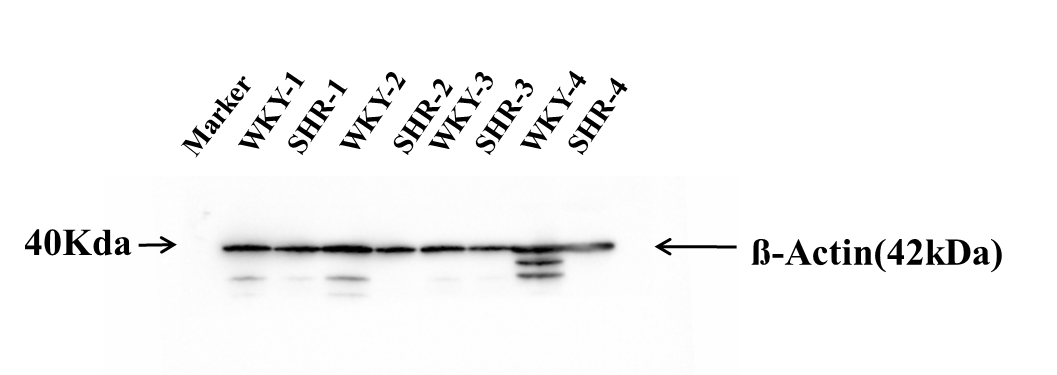


**Supplemental Figure 5.** Western blot analysis of Actin in the submandibular glands in SHR and WKY rats.
